# The impact of blood pressure variability and African Caribbean ethnicity on the progression of diabetic kidney disease in type 1 diabetes

**DOI:** 10.1007/s00125-026-06715-4

**Published:** 2026-04-01

**Authors:** Murat Ozdede, Panagiotis Pavlou, Valson Krasniqi, Kleoniki I. Athanasiadou, Salma Ayis, Stephen Thomas, Janaka Karalliedde

**Affiliations:** 1 School of Cardiovascular and Metabolic Medicine and Sciences, King’s College London, London, UK; 2 Faculty of Medicine, Department of Internal Medicine, Hacettepe University, Ankara, Turkey; 3 Department of Diabetes and Endocrinology, Guy’s and St Thomas’ NHS Foundation Trust, London, UK

**Keywords:** African Caribbean ethnicity, Blood pressure variability, Diabetic kidney disease, Type 1 diabetes

## Abstract

**Aims/hypothesis:**

Blood pressure variability (BPV) is a risk factor for kidney disease progression in hypertension and type 2 diabetes; however, the role of BPV in kidney disease in type 1 diabetes is unknown. The aim of this study was therefore to determine whether BPV has an impact on kidney disease progression in an ethnically diverse cohort of people with type 1 diabetes.

**Methods:**

We studied 3079 people (median age 36 [range 18–85] years; 50% female; 78.5% White, 10.9% African Caribbean, 4.5% Asian, 6.1% Other) with type 1 diabetes and baseline eGFR >45 ml/min per 1.73m^2^ attending two university hospital clinics between 2004 and 2018. BPV was assessed using visit-adjusted standard deviation (adj-SD), CV and average real variability (ARV) for systolic blood pressure (SBP) and diastolic blood pressure (DBP). The primary endpoint was eGFR decline of ≥50% from baseline with final eGFR <30 ml/min per 1.73m^2^, with death as a competing risk.

**Results:**

Over a 14 year period, 272 people (8.8%) reached the primary endpoint. All BPV metrics for SBP and DBP were significantly associated with the primary endpoint. ARVs of SBP and DBP and African Caribbean ethnicity emerged as risk factors in multivariable analyses, independent of traditional risk factors, including baseline blood pressure, eGFR, HbA_1c_, albuminuria and mean blood pressure in the exposure window. The strongest association was observed for the ARV of SBP (HR 1.54, 95% CI 1.27, 1.87, *p*<0.001).

**Conclusions/interpretation:**

In people with type 1 diabetes, SBP and DBP variability are associated with kidney disease progression. Further studies are needed to investigate if BPV is a modifiable risk factor for kidney disease progression.

**Graphical Abstract:**

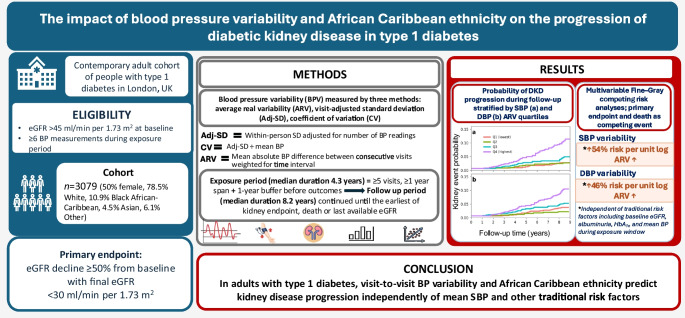

**Supplementary Information:**

The online version contains peer-reviewed but unedited supplementary material available at 10.1007/s00125-026-06715-4.


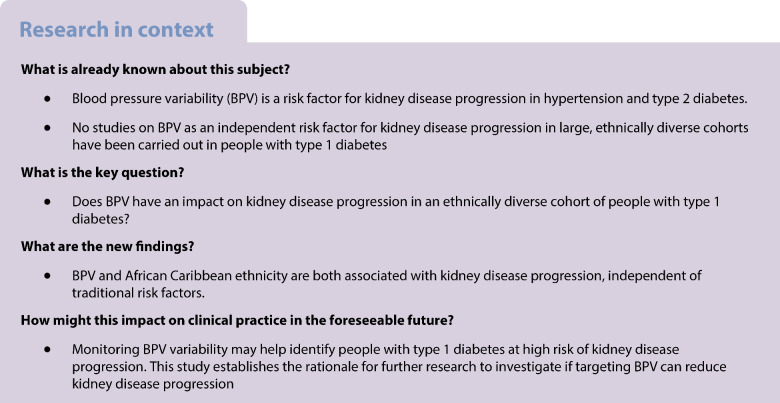


## Introduction

Diabetes is a leading cause of end-stage kidney disease (ESKD) and the lifetime risk of developing ESKD among people with type 1 diabetes is approximately 10–15% [1]. Despite major improvements in care in recent decades, chronic kidney disease (CKD) is a common complication of type 1 diabetes, affecting 30–40% of all individuals. People with type 1 diabetes and diabetic kidney disease (DKD) are at high risk of ESKD and cardiovascular events [2, 3].

Blood pressure (BP) is a key determinant of DKD [4, 5] and hypertension often drives its rapid progression [6]. Ethnicity has emerged as an important factor affecting BP control in people with hypertension with or without type 2 diabetes, with people of African Caribbean ethnicity being disproportionately affected [7, 8].

Emerging evidence suggests that increased blood pressure variability (BPV), indicated by visit-to-visit fluctuations in BP measurements, may cause kidney damage and CKD progression, measured by decline in eGFR or increase in urinary albumin, in people with type 2 diabetes [9]. More specifically, systolic BPV increments of 1 SD have been associated with an 8% higher risk of DKD progression and onset of ESKD, independent of baseline eGFR and urinary albumin excretion, in an ethnically diverse cohort of people with type 2 diabetes aged 30–70 years (62% White, 14% Black, 10% Hispanic, 14% Other) [9]. However, no impact of ethnicity on BPV-related progression of DKD was observed in this study.

BPV induces endothelial dysfunction leading to impaired autoregulation of renal blood flow and increased glomerular pressure, thereby accelerating renal function decline. Furthermore, frequent fluctuations in BP can increase mechanical stress on glomeruli promoting inflammation, fibrosis and scarring [10].

BPV has been previously implicated as a risk factor for DKD progression in two studies in people with type 1 diabetes with relatively small sample sizes [11, 12]. However, evidence remains sparse on the role of BPV as an independent predictor of DKD progression in large, ethnically diverse type 1 diabetes cohorts.

The majority of studies that have explored kidney endpoints or the general impact of BP in type 1 diabetes have included only people of White ethnicity [13, 14]. African Caribbean ethnicity increases the risk of DKD progression in people with type 1 diabetes, an effect that is independent of traditional risk factors [15]. The mechanism for this remains unclear. A previous study in healthy, normotensive individuals has demonstrated that people of Black heritage had significantly higher BPV than White individuals (*p*<0.05) [16].

There is scarcity of evidence regarding the impact of BPV and ethnicity on DKD progression in people with type 1 diabetes. The aim of this study was to determine whether there was a significant association between BPV, ethnicity and DKD progression in people with type 1 diabetes from an ethnically diverse population.

## Methods

### Study population

The dataset analysed was derived from a pre-existing, curated cohort of 5733 adults with type 1 diabetes of whom 50.7% were female, 13.4% were African Caribbean and 86.6% were non-African Caribbean. With regard to ethnicity and sex the study population was representative of an urban-dwelling cohort attending routine outpatient diabetes clinics between 2004 and 2018 at two large university hospitals in south London [15]. As part of the original cohort construction, individuals with pregnancy and documented non-diabetic CKD were excluded. This retrospective study used anonymised routine clinical data accessed by the clinical care team and was conducted in accordance with local audit protocols and hospital data governance committee approvals. Extracted information included date of birth, self-reported ethnicity and sex, systolic blood pressure (SBP) and diastolic blood pressure (DBP), laboratory measurements (serum creatinine, urine albumin/creatinine ratio [ACR], HbA_1c_ and lipid profile) and anthropometric measurements, recorded at each visit using standardised clinical protocols. Measurements from acute hospital admissions were excluded.

For our data analysis, we first identified all adults with type 1 diabetes and at least three valid eGFR measurements and a baseline eGFR ≥45 ml/min per 1.73 m^2^. We then excluded individuals with age at baseline below 18 years, fewer than five valid outpatient BP measurements, conflicted or noisy data or insufficient follow-up data. For each participant we defined baseline laboratory and clinical covariates using measurements recorded within a ±2 year window around the baseline eGFR date, selecting the value closest to the index date when multiple measurements were available. When multiple measurements were available for the same variable within this window, we used the value closest in time to the baseline eGFR date. If no measurement was available within the prespecified window, the variable was considered missing for that participant. Critically, the first BP measurement used for BPV estimation was required to occur after the latest date of the selected baseline covariate measurements, ensuring complete temporal separation between baseline characterisation and the BPV exposure period.

Missing baseline data were limited to lipid variables (5–8% for each lipid parameter). We performed multiple imputation by chained equations using the multivariate imputation by chained equations (mice) package in R with 20 imputed datasets, predictive mean matching and ten iterations per imputation. Imputation models included all baseline covariates plus the event indicator and follow-up time. Serum creatinine, eGFR, BP, HbA_1c_, ACR, age, sex and ethnicity were complete or required for cohort entry and were not imputed. Complete-case sensitivity analysis confirmed the consistency of the findings (electronic supplementary material [ESM] Table 1). Details of participant selection, including the number of individuals screened, excluded at each step and included in the final analysis, are shown in ESM Fig. 1.

Socioeconomic status was assessed using the Index of Multiple Deprivation (IMD), based on UK Office for National Statistics data and individual postcodes, stratified into national deciles (1 = most deprived, 10 = least deprived) [17].

### BP measurement

BP was measured using automated sphygmomanometers in the seated position on the non-dominant arm, with multiple readings taken per each outpatient clinic visit according to local hospital protocols; mean SBP and DBP were recorded in electronic health records. Serum creatinine was measured centrally and used to calculate eGFR using the updated CKD-EPI equation.

The BP devices used in outpatient diabetes clinics over the period of our study included the Welch Allyn LIFESIGN 52000/SPOT 4200B and Fukuda Denshi DS-8100N. Devices were calibrated annually by the medical equipment management service in accordance with hospital quality control procedures. This process includes visual inspection and a range of functional tests to verify accuracy, safety and functionality. Unit pressure tests are conducted at multiple setpoints (0, 50, 150, 250 mmHg) and results are compared against strict specifications. Voltage and current tests ensure that devices operate within acceptable electrical limits. Leak, dump and inflation tests evaluate the system’s ability to maintain and release pressure appropriately. Overpressure tests ensure safety by confirming that a device can withstand high pressure without malfunctioning. Button and interface tests verify that user controls and communication ports work properly. The electrical safety of monitors was also inspected.

### Exposure window and follow-up

To reduce bias from using BP measurements obtained close to the renal endpoint, BPV was estimated within a predefined exposure window before the start of outcome follow-up. For each participant, we considered all eligible outpatient BP measurements over a period of at least 1 and up to 5 years, requiring a minimum of five measurements within this window. In individuals who experienced DKD progression, only BP values recorded at least 1 year before the event were used to define the exposure window; subsequent measurements were not included in BPV estimation. Follow-up for kidney outcomes then commenced at the end of the BPV exposure window and continued until the earliest of DKD progression, death or the last available eGFR measurement.

### BPV metrics

BPV was assessed using three metrics for both SBP and DBP within the exposure window:

1. visit-adjusted standard deviation (adj-SD), calculated as the within-person SD (σ) adjusted for the number of BP readings (*n*):
  $$\sigma \times \sqrt{\frac{n-1}{2n}}$$
2. CV, defined as adj-SD divided by mean BP:
  $$\frac{adjSD}{\overline{x} }$$
3. time-normalised average real variability (ARV), computed as the mean absolute difference between successive BP readings (|*x*_*i*+1_–*x*_*i*_|), with weighting for the time interval (*t*_*i*_) between visits to account for irregular follow-up:
  $$\frac{1}{N-1}\sum_{i=1}^{N-1} \frac{\left|{x}_{i+1}-{x}_{i}\right|}{\mathrm{max}(ti+1-ti, 0.08)}$$

All variability metrics have their own distinct strengths and limitations. SD is the most commonly used measure of BPV because of its simplicity and ease of interpretation. However, SD is highly sensitive to extreme values and is strongly correlated with mean BP, limiting its ability to discriminate true variability from differences in mean BP levels. In addition, SD is substantially influenced by acute stressors, circadian BP changes and day–night differences, including nocturnal dipping patterns, which may inflate estimates of variability independently of short-term BP fluctuations [18–20].

To address the dependency of SD on mean BP, the CV expresses variability as a proportion of the mean BP. By partially correcting for the direct proportionality between mean BP and its variability, CV allows more meaningful comparisons across individuals with differing BP levels. Nonetheless, CV remains influenced by acute behavioural or environmental stressors and by circadian BP variation and, although its correlation with mean BP is weaker than that of SD, it is not entirely eliminated [18, 21, 22].

Both SD and CV share important limitations, including their inability to account for the temporal sequence of BP measurements. As a result, these indices may overestimate variability when BP changes occur gradually, such as during the transition between daytime and nighttime periods, rather than reflecting true short-term instability [20, 23].

The ARV has been proposed as an alternative metric that overcomes some of these limitations. ARV is calculated as the mean of the absolute differences between consecutive BP readings and explicitly incorporates the order of measurements, thereby providing a more direct assessment of short-term BP fluctuations. ARV is less affected by the frequency of BP measurements and, as it does not scale directly with the magnitude of BP values, its dependence on mean BP is substantially reduced. These features make ARV a potentially more robust marker of clinically relevant BP variability [18, 22, 23].

ARV has been shown to outperform SD [22]. In its basic formulation ARV assumes approximately equal spacing between measurements [22, 24]. To address this limitation, modified, time-sensitive extensions of ARV have been proposed [25]. In our study, we applied this approach and defined ARV as the mean absolute difference between consecutive BP measurements divided by the time between visits, with a lower bound imposed on Δt to avoid numerical instability for very short intervals. This construction places relatively greater weight on steeper changes over shorter time spans while still preserving the successive difference framework of mean successive variability.

For the primary analysis, ARV was selected as the main BPV metric based on its time-sensitive nature and prior literature on BPV [22, 26, 27]. To ensure reliable variability estimates, BPV metrics were log-transformed where skewed and subsequently standardised as *z* scores before inclusion in regression models.

### Endpoint definition

The primary endpoint was progression of DKD, defined as a sustained ≥50% decline in eGFR from baseline and eGFR <30 ml/min per 1.73 m^2^ based on two measurements. In participants who appeared to meet this threshold, a subsequent eGFR measurement within 12 months was required to confirm the decline. Death before DKD progression was treated as a competing risk in all Fine–Gray models, but individuals were censored at death in descriptive analyses and Cox models.

### Statistical analysis

Continuous variables are summarised as mean ± SD or median (IQR), as appropriate; categorical variables are described using frequencies and percentages. Between-group differences were assessed using *t* tests or Mann–Whitney *U* tests for continuous variables and χ^2^ tests for categorical variables.

For time-to-event analyses, we first used Cox proportional hazards models to examine univariable associations between each BPV metric, baseline covariates and DKD progression. To identify the most informative BPV metric, we fitted separate Cox models for adj-SD, CV and ARV for both SBP and DBP, and calculated Harrell’s concordance index (C-index) for each model to quantify discriminative performance, indicating how well the model predicts outcomes. Model discrimination was assessed with optimism correction through bootstrap internal validation (B=200 resamples). This comparison did not demonstrate superiority for any single BPV metric; ARV was selected due to its ability to capture time-dependent fluctuations between consecutive BP measurements, including irregular visit intervals [22].

We then assessed whether adding the ARV of SBP and DBP improved discrimination beyond conventional risk factors. Multivariable Cox models were built including age, baseline eGFR, HbA_1c_, log-transformed ACR, ethnicity and mean SBP during the BPV exposure window. We compared optimism-corrected C-indices across these specifications and confirmed that the inclusion of ARV metrics improved model discrimination. As a sensitivity analysis, we examined the functional form of the association between SBP variability and kidney outcomes using restricted cubic splines. We fitted Cox models with the ARV of SBP entered as a restricted cubic spline with four knots, adjusting for baseline covariates including SBP. Wald tests for the spline terms and likelihood ratio tests comparing linear vs spline parameterisations were used to assess evidence of non-linearity. Based on these results, final multivariable Fine–Gray competing risk regression models were constructed, incorporating ARV alongside relevant clinical covariates, with death as the competing event. To assess effect modification by ethnicity, we additionally fitted models including an interaction term between ethnicity (African Caribbean vs non-African Caribbean) and BPV.

For visualisation, we compared progression across quartiles of BPV using Kaplan–Meier failure probability plots and derived cumulative incidence functions for DKD progression and death to illustrate the competing risk framework. All analyses were conducted using R version 4.4.2 (R Foundation for Statistical Computing, Vienna, Austria).

## Results

The baseline cohort characteristics (*n*=3079, 50% female) are summarised in Table 1. The median (range) age was 36 (18–85) years, and the median (range) duration of diabetes was 13 (0–65) years. The majority of participants (89.1%) were non-African Caribbean (78.5% White, 4.5% Asian, 6.1% Other); 10.9% were of African Caribbean ethnicity. Mean (±SD) baseline SBP and DBP were 124.1 (±15.2) mmHg and 74.1(±8.9) mmHg, respectively. Mean SBP and DBP over the exposure window were 124.4 (±11.6) and 73.6 (±6.8) mmHg, respectively. Baseline eGFR was 89.3 (±22.7) ml/min per 1.73m^2^, with a serum creatinine level of 75.4 (±16.6) mmol/l. Participants had a BMI of 25.6 (±4.6) kg/m^2^, total cholesterol level of 4.7 (±1.0) mmol/l, HDL-cholesterol level of 1.6 (±0.5) mmol/l, LDL-cholesterol level of 2.5 (±0.8) mmol/l, triglyceride level of 1.2 (±0.9) mmol/l and mean log-transformed ACR of 2.6 (±1.2) mg/mmol. These results were consistent with our previous observations [15]. Our BPV exposure window approach yielded a median of eight BP measurements (IQR 6–10) over a median exposure span of 4.2 years (IQR 3.1–5.0).

**Table 1:** Baseline clinical and biochemical characteristics of the total cohort with type 1 diabetes

Variable	Total cohort (*n*=3079)
Female sex	1539 (50.0)
Age (years)	36.0 [18.0–85.0]
Ethnicity	
African Caribbean	336 (10.9)
Non-African Caribbean	2743 (89.1)
Serum creatinine (mmol/l)	75.4±16.6
Type 1 diabetes duration (years)	13.0 [0–65.0]
BMI (kg/m^2^)	25.6±4.6
eGFR (ml/min per 1.73 m^2^)	89.3±22.7
Weight (kg)	74.2±15.3
Baseline SBP (mmHg)	124.1±15.2
Baseline DBP (mmHg)	74.1±8.9
Mean SBP in exposure window (mmHg)^a^	124.4±11.6
Mean DBP in exposure window (mmHg)^a^	73.6±6.8
HbA_1c_ (mmol/mol)	72.0±23.3
HbA_1c_ (%)	8.7± 2.1
Total cholesterol (mmol/l)	4.7±1.0
HDL-cholesterol (mmol/l)	1.6±0.5
LDL-cholesterol (mmol/l)	2.5±0.8
Triglycerides (mmol/l)	1.2±0.9
Urine ACR (mg/mmol)	13.0 [5.0–43.0]
IMD decile^b^	3.0 [2.0–5.0]

Continuous variables are presented as mean ± SD or median [IQR]. Categorical variables are presented as *n* (%)

^a^Mean SBP and DBP were calculated from the means of all values per participant during the exposure period

^b^IMD is ranked from 1 [most deprived] to 10 [least deprived])

A total of 272 people (8.8%) reached the primary renal endpoint with death as a competing risk as demonstrated in the cumulative incidence plot (ESM Fig. 2); those with the highest BPV had the greatest number of events. Those who reached the endpoint were significantly older with higher baseline HbA_1c_, baseline SBP, mean SBP during exposure, triglyceride levels and urine ACR than those who did not (Table 2). A summary of the BPV metrics for the whole cohort is provided in ESM Table 2. The univariate analysis presented in ESM Table 3 revealed that all BPV metrics (adj-SD, CV, ARV) for SBP and DBP were significantly associated with the primary endpoint.

**Table 2:** Comparison of baseline clinical and biochemical characteristics between those who did and those who did not reach the primary endpoint of eGFR decline ≥50% from baseline with a final eGFR <30 ml/min per 1.73 m^2^ (*n*=3079)

Variable	No event^a^	Event^b^	*p* value
Male	1475 (52.5)	122 (44.9)	0.45
Age (years)	38.2±13.5	48.5±15.9	<0.001
African Caribbean	305 (10.9)	31 (11.4)	<0.001
Serum creatinine (mmol/l)	75.0±16.5	83.1±16.3	<0.001
Type 1 diabetes duration (years)	14.7±12.1	18.2±14.7	0.005
Weight (kg)	74.3±15.2	73.5±16.9	0.59
BMI (kg/m^2^)	25.5±4.6	26.4±5.2	0.05
eGFR (ml/min per 1.73 m^2^)	90.0±22.6	75.2±21.4	<0.001
Baseline SBP (mmHg)	123.9±15.1	128.9±17.4	<0.001
Baseline DBP (mmHg)	74.0±8.9	74.3±9.7	0.79
Mean SBP during exposure period (mmHg)^c^	124.1±11.4	131.3±13.5	<0.001
Mean DBP during exposure period (mmHg)^c^	73.6±6.7	73.8±8.0	0.69
HbA_1c_ (mmol/mol)	71.5±22.8	82.2±29.2	<0.001
HbA_1c_ (%)	8.7±2.1	9.7±2.7	<0.001
HDL-cholesterol (mmol/l)	1.6±0.5	1.6±0.5	0.94
LDL-cholesterol (mmol/l)	2.5±0.8	2.5±0.9	0.11
Total cholesterol (mmol/l)	4.7±0.9	4.8±1.3	0.28
Triglycerides (mmol/l)	1.2±0.9	1.6±1.3	0.003
Log-transformed ACR (mg/mmol)	2.6±1.2	3.1±1.4	<0.001
IMD decile^d^	3.0 [2.0–5.0]	3.0 [2.0–5.0]	0.87
adj_SBP	6.1±2.3	8.6±3.0	<0.001
adj_DBP	3.9±1.4	4.9±1.9	<0.001
cv_SBP	0.1±0.02	0.1±0.02	<0.001
cv_DBP	0.1±0.02	0.1±0.02	<0.001
arv_SBP	30.4±21.1	37.1±31.3	<0.001
arv_DBP	20.1±14.1	26.2±17.9	<0.001

Continuous variables are presented as mean ± SD or median [IQR]. Categorical variables are presented as *n* (%)

^a^No event = participants with type 1 diabetes who did not experience the primary endpoint of kidney function decline (*n*=2807)

^b^Event = participants with type 1 diabetes who reached the primary endpoint (*n*=272)

^c^Mean SBP and DBP were calculated from the means of all values per participant during the exposure period

^d^IMD is ranked from 1 (most deprived) to 10 (least deprived)

The C-index values for adj-SD, CV and ARV for SBP and DBP were calculated using univariate Fine–Gray competing risk models with death as the competing event (ESM Table 4). Values were similar across variability metrics with no clear indication of superiority for any single metric.

We compared multivariable Cox proportional hazards models with and without BPV metrics, adjusting for the same baseline covariates (SBP, ethnicity, baseline eGFR, HbA_1c_, log ACR, triglycerides). For SBP we tested both baseline SBP (single measurement at cohort entry) and mean SBP during the exposure period (average of all measurements used to calculate BPV). We found that mean SBP during the exposure period outperforms baseline SBP and is a significantly stronger predictor of DKD progression. Models including mean SBP also demonstrated superior discrimination (C-index 0.772 vs 0.768) and fit (Akaike’s information criterion [AIC] 1968.1 vs 1979.8, ΔAIC=–11.7) (ESM Table 5). We therefore retained mean SBP in all subsequent models as it better captures SBP exposure over the same time frame as BPV assessment.

Multivariable hazard models incorporating baseline covariates with BPV indices demonstrated significant improvements in model performance metrics (ESM Table 5). While baseline risk factors, including higher age, lower eGFR, African Caribbean ethnicity, higher HbA_1c_, higher mean SBP during exposure and elevated ACR levels, were strongly associated with the primary endpoint, the models incorporating the log-adjusted ARV of SBP and log-adjusted ARV of DBP exhibited a higher C-index (0.798 and 0.796, respectively) than the baseline model (0.772), indicating superior overall performance when these BPV metrics were included in the model.

**Fig. 1:**
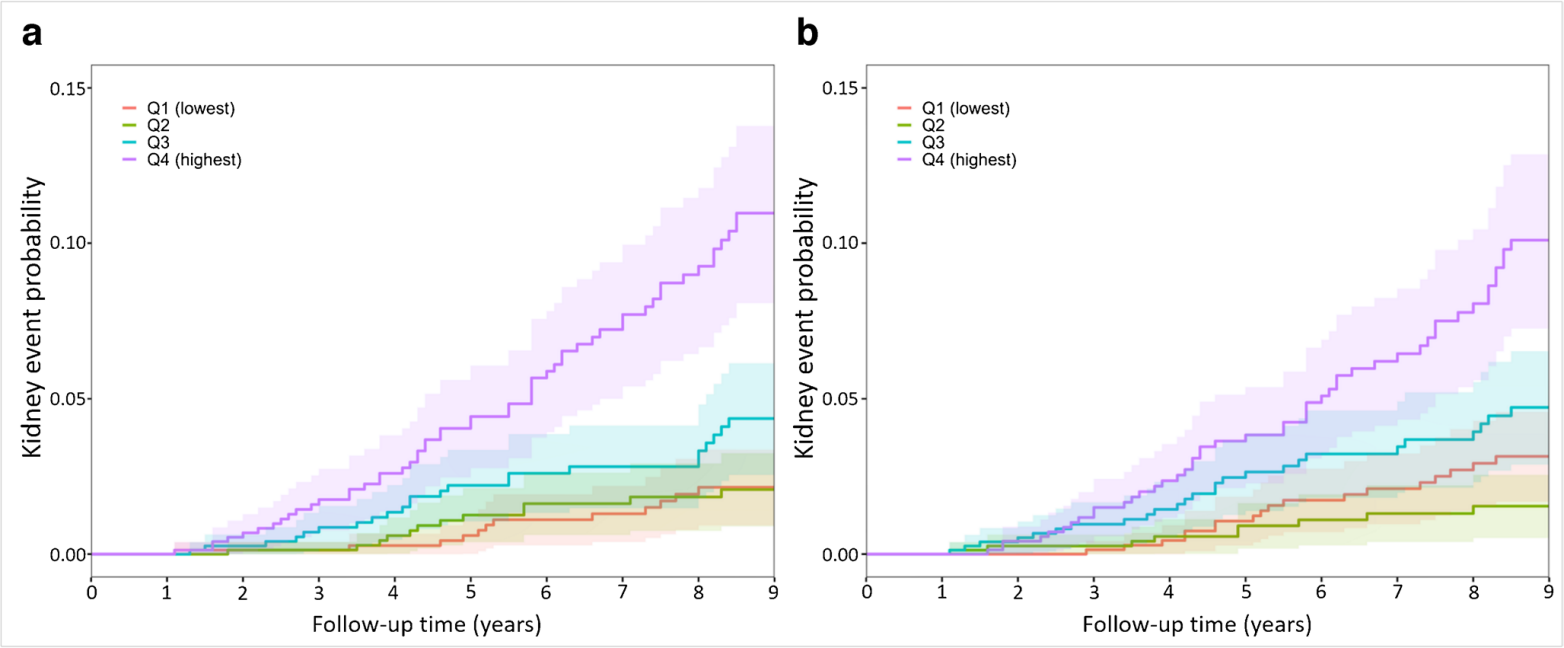
Kaplan–Meier failure probability plots showing kidney event probability stratified by BPV quartiles based on ARV of (**a**) SBP and (**b**) DBP (*n*=3079; *n*=144 kidney events)

Multivariate Fine–Gray models (Table 3) treating death as a competing risk identified a significant association between the log-adjusted ARV of SBP (HR 1.54, 95% CI 1.27, 1.87, *p*<0.001) and log-adjusted ARV of DBP (HR 1.46, 95% CI 1.21, 1.76, *p*<0.001) and DKD progression. These results indicate that there is an approximately 50% increase in risk of DKD progression as defined by the primary endpoint per 1 point increase in log-adjusted ARV of SBP and log-adjusted ARV of DBP. This association was independent of other clinically relevant risk factors such as age, HbA_1c_, SBP, urine ACR and BMI. The risk of DKD progression over time across BPV quartiles is visualised in Fig. 1, highlighting clear differences between quartiles, with those in the highest BPV quartile demonstrating the greatest risk.

**Table 3:** Multivariate Fine–Gray models examining the effect of baseline characteristics and BPV indices on the primary kidney outcome (eGFR decline ≥50% from baseline with a final eGFR <30 ml/min per 1.73 m^2^)

Variable	Model 1: baseline			Model 2: log ARV SBP addition			Model 3: log ARV DBP addition		
	HR	*p* value	95% CI	HR	*p* value	95% CI	HR	*p* value	95% CI
Age (years)	1.03	<0.001	1.01, 1.04	1.02	0.001	1.01, 1.04	1.03	<0.001	1.01, 1.04
eGFR (ml/min per 1.73 m^2^)	0.99	0.05	0.98, 1.00	0.99	0.03	0.98, 1.00	0.99	0.03	0.98, 1.00
HbA_1c_ (mmol/mol)	1.02	<0.001	1.02, 1.03	1.02	<0.001	1.01, 1.03	1.02	<0.001	1.01, 1.03
Log-transformed ACR (mg/mmol)	1.26	0.001	1.1, 1.46	1.26	0.002	1.09, 1.45	1.26	0.001	1.09, 1.45
Mean SBP (mmHg)^a^	1.03	<0.001	1.01, 1.04	1.02	0.009	1.01, 1.04	1.02	0.004	1.01, 1.04
Ethnicity: African Caribbean	1.67	0.018	1.11, 2.53	1.60	0.025	1.06, 2.43	1.64	0.02	1.09, 2.48
ARV SBP (log transformed) (mmHg)				1.54	<0.001	1.27, 1.87			
ARV DBP (log transformed) (mmHg)							1.46	<0.001	1.21, 1.76

^a^Mean SBP was calculated from values during the exposure period

African Caribbean ethnicity was significantly associated with DKD progression in all multivariate Fine–Gray models (HR 1.60–1.67, *p*<0.05 for all models). There was no evidence that the association between BPV and kidney outcomes differed by African Caribbean ethnicity (ethnicity and systolic BPV interaction *p*=0.23, 95% CI 0.87, 1.84; ethnicity and diastolic BPV interaction *p*=0.59, 95% CI 0.85, 2.04).

Restricted cubic spline models did not provide strong evidence of non-linearity in the association between BPV and kidney outcomes (for ARV of SBP, non-linear χ^2^=3.3 on 2 df, *p*=0.19; likelihood ratio test linear vs spline *p*=0.21). The exposure–response curve was approximately flat at low variability and increased monotonically at higher levels of variability, supporting the use of standardised linear ARV terms in the main models (ESM Fig. 3).

## Discussion

In a large ethnically diverse cohort of people with type 1 diabetes we observed a significant relationship of BPV and African Caribbean ethnicity with DKD progression that was independent of traditional risk factors. We also found that the impact of BPV and African Caribbean ethnicity persisted in competing risk models where death was a competing event with the DKD progression outcome. In addition, BPV was significantly associated with DKD progression independently of mean BP over the exposure period, highlighting that achieving both BP targets and BP stability may be important for kidney protection in type 1 diabetes.

Recent evidence has linked BPV to an increased risk of ESKD in people with CKD stages 1–4, type 2 diabetes, hypertension and non-diabetic CKD [26–29]. This association is most relevant for ESKD incidence [30], with more conflicting results observed for the association between BPV and eGFR decline or increase in serum creatinine (18–21). The effect of BPV on kidney function was observed in two studies that used an eGFR decline >3 ml/min per 1.73 m^2^ per year (Korean population; *n*=470; 90.4% hypertensive; 46.6% diabetes; CKD stages 3–5; mean±SD age 60.9±12.0 years; 55.1% male) [31] and a decrease in eGFR to <45 ml/min per 1.73 m^2^ (Japanese population; *n*=664; type 2 diabetes; mean±SD age 55.7±9.2 years; 82% male) as endpoints [32]. However, the pooled effect of BPV on eGFR decline or increase in serum creatinine was not significant in a recent meta-analysis [33].

The majority of previous studies included predominantly White, Asian and male populations aged >60 years with type 2 diabetes, hypertension or other causes of CKD [33] and used various definitions of kidney function decline. However, in contrast to our study, competing risk analyses were not performed, which is an important consideration given that CKD per se is a risk factor for premature mortality.

There is a paucity of evidence on the role of BPV in type 1 diabetes and the impact of ethnicity on the relationship between BPV and DKD-related kidney function decline. In our study, in a cohort of people with type 1 diabetes (median age 36 years) followed over 14 years, we observed significant differences between individuals who reached the primary kidney outcome and those who did not. Notably, older individuals with higher HbA_1c_ levels, albuminuria and lower eGFR at baseline were at increased risk of DKD progression, while African Caribbean ethnicity was also associated with a higher risk for DKD progression, consistent with the existing literature [15, 34–36].

Our results demonstrate that the BPV indices adj-SD, CV and ARV are significantly associated with DKD progression independently of traditional risk factors in individuals with type 1 diabetes. The effect size for DKD progression risk due to BPV that we observed in our study (HR 1.54 for ARV of SBP, HR 1.46 for ARV of DBP) was higher than that observed by McMullan et al (HR 1.08) but lower than the effect sizes reported by Jhee et al (HR 1.68) and Takao et al (HR 2.43). However, comparability with these studies is limited due to significant differences in baseline risk, cohort characteristics, BPV metrics, endpoint definitions and duration of follow-up [9, 31, 32].

Our study is the first to report an independent role of BPV and ethnicity in DKD progression in people with type 1 diabetes. A previous study of 1062 individuals with type 1 diabetes showed an association between SBP variability and eGFR decline; however, the authors did not report on ethnicity, the sole method used to assess SBP variability was SD of the residuals in individual linear regression models, and death was not incorporated as a competing risk [12]. In addition, there was an absence of a minimum temporal separation between the last BP measurement of the exposure window and onset of a renal endpoint, which may have introduced confounding. A study by Rotbain Curovic et al investigated the impact of BPV and other variables on DKD progression and included a proportion of Black individuals (9%) with type 1 diabetes; however, it had several notable limitations [11]. Specifically, it did not examine the independent impact of BPV, as it was incorporated only as part of a risk cluster composed of multiple parameters. Additionally, the study was a post hoc analysis of the Preventing Early Renal Loss in Diabetes (PERL) trial with a relatively small sample size (*n*=404). Notably, both of these studies used baseline SBP (based on single-visit BP values) as a covariate in the multivariate models and did not include mean SBP over the entire exposure window, thereby not assessing the impact of BPV independently of mean SBP exposure.

A study in type 2 diabetes demonstrated that higher SBP variability was associated with a 49% higher risk of DKD development [37]. Moreover, a post hoc analysis of two clinical trials revealed that SBP variability was independently linked to adverse kidney outcomes (defined as time to confirmed doubling of serum creatinine level, end-stage renal disease or death) in type 2 diabetes [9]. Additionally, the Antihypertensive and Lipid-Lowering Treatment to Prevent Heart Attack Trial (ALLHAT) indicated a graded relationship, showing that greater visit-to-visit BPV was associated with an elevated risk of renal complications (defined as incident ESKD after assessment or ≥50% decline in eGFR between 24 months and 48 or 72 months after randomisation), independent of mean BP levels [38].

The underlying mechanisms associated with BPV and DKD progression are not fully understood. A number of mechanisms have been proposed including associations of BPV with renal vascular resistance [39], oscillatory endothelial shear stress [40, 41], impact of renal autoregulatory responses [42] and ischaemia–reperfusion-related kidney injury [43].

Other mechanisms that have been associated with BPV and may be implicated in DKD progression include increased arterial stiffness and endothelial dysfunction [44–46]. Variability of SBP and DBP was associated with increased arterial stiffness in a study of individuals with untreated hypertension while, in another study examining the cohort from the Multi-Ethnic Study of Atherosclerosis, higher BPV was associated with decreased aortic distensibility [45, 46].

In the context of DKD, an increase in sympathetic nervous system stimulation plays an important role and may influence the association between increased BPV and DKD progression through multiple cellular, structural and functional mechanisms [47, 48]. Similar overactivation of the inflammatory cascade driven by the renin–angiotensin–aldosterone system is noted in DKD, and related end-organ damage, such as increased left ventricular mass, may be influenced by BPV [49]. In parallel, some data from human and animal studies exist that suggest a possible link between increased activity of the renin–angiotensin–aldosterone system and BPV [50].

Our results support further investigations to study these mechanisms but also to explore further the role of BPV and African Caribbean ethnicity in DKD progression. However, population membership per se may not be the only factor in determining propensity to DKD progression, as there may be a confluence of genetic, environmental, sociocultural and policy factors that could contribute to risk and further studies to identify causal risk factors are needed.

We did not observe a statistically significant interaction between ethnicity and the effect of BPV on DKD progression. While the present study was not designed to investigate underlying mechanisms, several biological and contextual factors may contribute to the observed disparity in people of African Caribbean ethnicity. Genetic susceptibility related to African Caribbean ethnicity, including the higher prevalence of *APOL1* risk variants, has been strongly linked to accelerated DKD progression in type 2 diabetes [51]. In addition, African Caribbean populations experience a higher burden of hypertension, salt sensitivity and BPV, all of which are plausible contributors to DKD progression [16, 52, 53]. Future mechanistic studies examining whether these physiological characteristics are more pronounced in African Caribbean adults with type 1 diabetes may help clarify the pathways underlying our observations.

Our study has several strengths and certain limitations. The study’s novelty lies in the inclusion of a large ethnically diverse cohort from a publicly funded healthcare system. The long follow-up period of 14 years provides a key advantage compared with previous studies. Further strengths include its contemporaneous real-world nature and the fact that all laboratory data and BP measures were performed using standardised processes. The use of multiple statistical models including competing risk analyses and concordance indexes may enhance the reliability of our conclusions. In addition, our cohort had a balanced sex distribution (50.0% female), thereby strengthening the external validity and generalisability of our findings. It is also worth noting that our study included a substantially higher representation of female participants than other studies in this area.

Limitations include the retrospective study design, which limits casual inferences, and cohort selection from two large London university hospitals, where individuals with complex type 1 diabetes are referred, with the associated risk of referral bias. A further major limitation is the lack of information on BP medication use and adherence, in particular the use of drugs such as renin–angiotensin–aldosterone inhibitors that may affect long-term BPV or kidney function. Moreover, in this study the diagnosis of type 1 diabetes was not confirmed through laboratory antibody testing but was instead based on medical record documentation from diabetes specialist clinics in tertiary centres. As a result, we cannot exclude the misclassification of some individuals. In addition, it is worth noting that we only included individuals with five or more BP measurements, which may limit generalisability in those with very sporadic clinic attendance; however, this threshold aligns with established methodological standards in this research field [54–57]. Reverse causality may be plausible in our results, as people with DKD progression may have greater BPV. However, to mitigate against this we temporally separated the kidney progression endpoint from BPV exposure by using exposure and follow-up periods. We also included mean SBP over the exposure window in our analysis rather than baseline SBP (based on a single value) to better capture BP exposure over the same time frame as BPV assessment. Finally, in our analysis we chose summary variability metrics that collapse repeated BP measurements into a single value instead of using joint longitudinal–survival models. Even though we acknowledge that the latter may represent a complementary analytical strategy that could provide additional insights into temporal relationships between BP evolution and kidney function decline, there are also limitations of such an approach due to convergence challenges, particularly when incorporating time-varying covariates and competing risk frameworks [58–60]. Furthermore, variability summary metrics can provide clinically interpretable measures that can be directly translated into clinical practice and patient counselling, and are therefore widely used in cardiovascular and renal outcome research [18, 22, 23].

In conclusion, this study highlights the key independent role of BPV and African Caribbean ethnicity in the progression of DKD among people with type 1 diabetes. Further studies are needed to investigate if BPV is a modifiable risk factor for kidney disease progression.

## Supplementary Information

Below is the link to the electronic supplementary material.

ESM (PDF 644 KB)

## Data Availability

The data that support the findings of this study are not openly available for reasons of sensitivity and are available from the corresponding author on reasonable request. Data are located in controlled access data storage at King’s College London.

## Abbreviations

ACR: Albumin/creatinine ratio
ARV: Average real variability
BP: Blood pressure
BPV: Blood pressure variability
CKD: Chronic kidney disease
DBP: Diastolic blood pressure
DKD: Diabetic kidney disease
ESKD: End-stage kidney disease
IMD: Index of Multiple Deprivation
SBP: Systolic blood pressure
