# Supplementary material for: The impact of blood pressure variability and African Caribbean ethnicity on the progression of diabetic kidney disease in type 1 diabetes
Source: Diabetologia. 2026 Apr 1;69(7):2061–72. doi: 10.1007/s00125-026-06715-4 (PMC13236763; doi:10.1007/s00125-026-06715-4)

ESM Table 1. Sensitivity Analysis: Multivariable Cox Models with Complete-Case Analysis (n=2,838)

Variable	Beta	HR	HR_CI	p_value
n= 2838, number of events= 139				
Complete case baseline model				
Age (years)	003	1.03	1.01-1.04	<0.001
eGFR (mL/min/1.73 m^2)	-0.01	0.99	0.98-1.00	0.013
HBA1C_baseline (mmol/mol)	0.02	1.02	1.02-1.03	<0.001
Log ACR (mg/mmol)	0.25	1.29	1.12-1.48	<0.001
Mean Systolic Blood Pressure (mmHg)	0.03	1.03	1.01-1.04	<0.001
Triglycerides	0.16	1.17	1.03-1.33	0.019
Ethnicity – African Caribbean	0.46	1.58	1.04-2.41	0.033
Complete case model with Log ARV SBP addition				
Age (years)	0.026	1.03	1.01-1.04	<0.001
eGFR (mL/min/1.73 m^2)	-0.01	0.99	0.98-1.00	0.01
HBA1C_baseline (mmol/mol)	0.02	1.02	1.01-1.03	<0.001
Log ACR (mg/mmol)	0.24	1.27	1.11-1.46	<0.001
Mean Systolic Blood Pressure (mmHg)	0.02	1.02	1.01-1.04	0.01
Triglycerides	0.11	1.12	0.98-1.27	0.09
Ethnicity – African Caribbean	0.42	1.53	1.00-2.33	0.05
ARV SBP (Log transformed) (mmHg)	0.43	1.53	1.27-1.84	<0.001
Complete case model with Log ARV DBP addition				
Age (years)	0.029	1.03	1.02-1.04	<0.001
eGFR (mL/min/1.73 m^2)	-0.01	0.99	0.98-1.00	0.01
HBA1C_baseline (mmol/mol)	0.02	1.02	1.02-1.03	<0.001
Log ACR (mg/mmol)	0.25	1.29	1.12-1.47	<0.001

Mean Systolic Blood Pressure (mmHg)	0.02	1.02	1.01-1.04	0.001
Triglycerides	0.12	1.13	0.99-1.29	0.07
Ethnicity – African Caribbean	0.43	1.53	1.00-2.34	0.05
ARV DBP (Log transformed) (mmHg)	0.40	1.49	1.25-1.78	<0.001

ESM Table 2. Summary of Blood Pressure Variability Metrics

Variability Metrics	Total Cohort mean (± SD)
**Standard Deviation of Systolic Blood Pressure (visit adjusted)**	6.29 ± 2.44
**Standard Deviation of Diastolic Blood Pressure (visit adjusted)**	4.03 ± 1.51
**Coefficient of Variation of Systolic Blood Pressure**	0.05 ± 0.02
**Coefficient of Variation of Diastolic Blood Pressure**	0.05 ± 0.02
**Average Real Variability of Systolic Blood Pressure**	31.52 ± 21.82
**Average Real Variability of Diastolic Blood Pressure**	20.63 ± 14.55

ESM Table 3. Univariate Analysis of Baseline Characteristics and Their Association with Primary Kidney Outcome (eGFR Decline ≥50% From Baseline with Final eGFR <30 mL/min/1.73 m²)

Variables	Coefficient	Hazard Ratio (HR)	95% Confidence Interval (CI)	p-value
**Age (Years)**	0.04	1.04	1.03, 1.05	<0.001
**BMI (kg/m²)**	0.03	1.03	1.00, 1.07	0.062
**eGFR (mL/min/1.73 m²)**	-0.03	0.97	0.96, 0.98	<0.001
**HbA1c_baseline (mmol/mol)**	0.02	1.02	1.01, 1.03	<0.001
**Log transformed urine ACR (mg/mmol)**	0.36	1.43	1.26, 1.64	<0.001
**Mean Systolic Blood Pressure in exposure window (mmHg)**	0.04	1.05	1.03, 1.06	<0.001
**Mean Diastolic Blood Pressure in exposure window (mmHg)**	0.02	1.02	0.99, 1.04	0.21
**Ethnicity (African Caribbean)**	0.84	2.32	1.15, 3.46	<0.001
**Triglycerides (mmol/L)**	0.25	1.28	1.15, 1.44	<0.001
**Diabetes Duration (Years)**	0.01	1.01	1.00, 1.02	0.081
Variability indices				
**Standard Deviation of Systolic Blood Pressure (visit adjusted)**	0.97	2.63	2.19, 3.18	<0.001
**Standard Deviation of Diastolic Blood Pressure (visit adjusted)**	0.59	1.81	1.50, 2.19	<0.001
**Coefficient of Variation of Systolic Blood Pressure**	0.72	2.05	1.78, 2.36	<0.001
**Coefficient of Variation of Diastolic Blood Pressure**	0.50	1.66	1.42, 1.93	<0.001
**Average Real Variability of Systolic Blood Pressure**	0.65	1.91	1.60, 2.29	<0.001
**Average Real Variability of Diastolic Blood Pressure**	0.48	1.62	1.36, 1.93	<0.001

Abbreviations: BMI – Body Mass Index; eGFR – Estimated Glomerular Filtration Rate; HbA1c – Haemoglobin A1c; Log ACR – Logarithmic Transformation of the Albumin-to-Creatinine Ratio (ACR); PP – Pulse Pressure; MAP – mean arterial pressure.

ESM Table 4. Concordance Index (C-Index) Values of Univariate Fine and Gray Models of Variability Indices

Univariate Models	C_index
1 Adj SD SBP	0.25
2 Adj SD DBP	0.29
3 ARV SBP	0.32
4 ARV DBP	0.33
5 CV SBP	0.27
6 CV DBP	0.31

ESM Table 5. Multivariate Cox Models with optimism corrected C-indices

	Baseline Model with Baseline Systolic Pressure as a covariate				Baseline Model with Mean Systolic Pressure as a covariate			
	**Beta**	**Hazard Ratio (HR)**	**95% Confidence Interval (CI)**	**p-value**	**Beta**	**Hazard Ratio (HR)**	**95% Confidence Interval (CI)**	**p-value**
Age (Years)	0.035658	1.036301	[1.02-1.05]	<0.001	0.029228	1.029659	[1.02-1.04]	<0.001
eGFR (mL/min/1.73 m2)	-0.0118	0.98827	[0.98-1.00]	0.023	-0.01116	0.988897	[0.98-1.00]	0.031
HBA1c_baseline (mmol/mol)	0.022858	1.023122	[1.02-1.03]	<0.001	0.022777	1.023038	[1.02-1.03]	<0.001
ACR (mg/mmol)†	0.252352	1.287049	[1.13-1.47]	<0.001	0.247451	1.280757	[1.12-1.46]	<0.001
Baseline Systolic Blood Pressure (mmHg)<br>Mean Systolic Blood Pressure (mmHg)	0.007821	1.007851	[1.00-1.02]	0.142	0.026448	1.026801	[1.01-1.04]	<0.001
Triglycerides	0.188858	1.20787	[1.06-1.37]	0.004	0.171546	1.187138	[1.04-1.35]	0.009
Ethnicity (African Caribbean)	0.545905	1.726171	[1.14-2.61]	0.01	0.497279	1.644241	[1.09-2.49]	0.018
C index†		0.768	[0.729-0.807]			0.772	[0.733-0.811]	
AIC		1979.8				1968.1		

	Systolic Variability Model with Baseline Systolic Pressure as a covariate				Systolic Variability Model with Mean Systolic Pressure as a covariate			
	**beta**	**Hazard Ratio (HR)**	**95% Confidence Interval (CI)**	**p_value**	**beta**	**Hazard Ratio (HR)**	**95% Confidence Interval (CI)**	**p_value**
Age (Years)	0.033055	1.033608	[1.02-1.05]	<0.001	0.027681	1.028067	[1.01-1.04]	<0.001
eGFR (mL/min/1.73 m2)	-0.01242	0.98766	[0.98-1.00]	0.015	-0.01167	0.9884	[0.98-1.00]	0.022
HBA1c_baseline (mmol/mol)	0.019335	1.019523	[1.01-1.03]	<0.001	0.019559	1.019752	[1.01-1.03]	<0.001
ACR (mg/mmol)†	0.239439	1.270537	[1.11-1.45]	<0.001	0.238281	1.269065	[1.11-1.45]	<0.001
Baseline Systolic Blood Pressure (mmHg)<br>Mean Systolic Blood Pressure (mmHg)	0.004279	1.004288	[0.99-1.01]	0.42	0.020462	1.020673	[1.01-1.04]	0.005
Triglycerides	0.138114	1.148107	[1.01-1.30]	0.033	0.12536	1.133557	[1.00-1.29]	0.057
Ethnicity (African Caribbean)	0.506127	1.658853	[1.10-2.51]	0.017	0.459931	1.583965	[1.05-2.40]	0.029
ARV of Systolic Blood Pressure†	0.478407	1.613501	[1.37-1.91]	<0.001	0.418425	1.519566	[1.27-1.82]	<0.001
C index†		0.796	(0.757-0.835)			0.798	(0.759-0.838)	
AIC		1956.12				1949		
**Diastolic Variability Model with Baseline Systolic Pressure as a covariate**					**Diastolic Variability Model with Mean Systolic Pressure as a covariate**			

	Beta	Hazard Ratio (HR)	95% Confidence Interval (CI)	p-value	Beta	Hazard Ratio (HR)	95% Confidence Interval (CI)	p-value
Age (Years)	0.036887	1.037576	[1.02-1.05]	<0.001	0.030942	1.031426	[1.02-1.05]	<0.001
eGFR (mL/min/1.73 m2)	-0.01278	0.987301	[0.98-1.00]	0.013	-0.01193	0.988145	[0.98-1.00]	0.02
HBA1c_baseline (mmol/mol)	0.020095	1.020298	[1.01-1.03]	<0.001	0.020457	1.020668	[1.01-1.03]	<0.001
ACR (mg/mmol)†	0.249155	1.282941	[1.12-1.47]	<0.001	0.243005	1.275074	[1.12-1.46]	<0.001
Baseline Systolic Blood Pressure (mmHg)<br>Mean Systolic Blood Pressure (mmHg)	0.005276	1.00529	[0.99-1.02]	0.32	0.022619	1.022877	[1.01-1.04]	0.001
Triglycerides	0.15671	1.169656	[1.03-1.33]	0.017	0.142846	1.153552	[1.01-1.31]	0.032
Ethnicity (African Caribbean)	0.525105	1.690636	[1.12-2.55]	0.013	0.471097	1.60175	[1.06-2.42]	0.025
ARV of Diastolic Blood Pressure†	0.406963	1.502248	[1.26-1.79]	<0.001	0.382124	1.465394	[1.23-1.74]	<0.001
C index†		0.792	(0.753-0.832)			0.796	(0.757-0.836)	
AIC		1960				1951.3		

†Optimism-corrected C-indices obtained through bootstrap internal validation (B=200 resamples).

ESM Figure 1. STROBE flow diagram

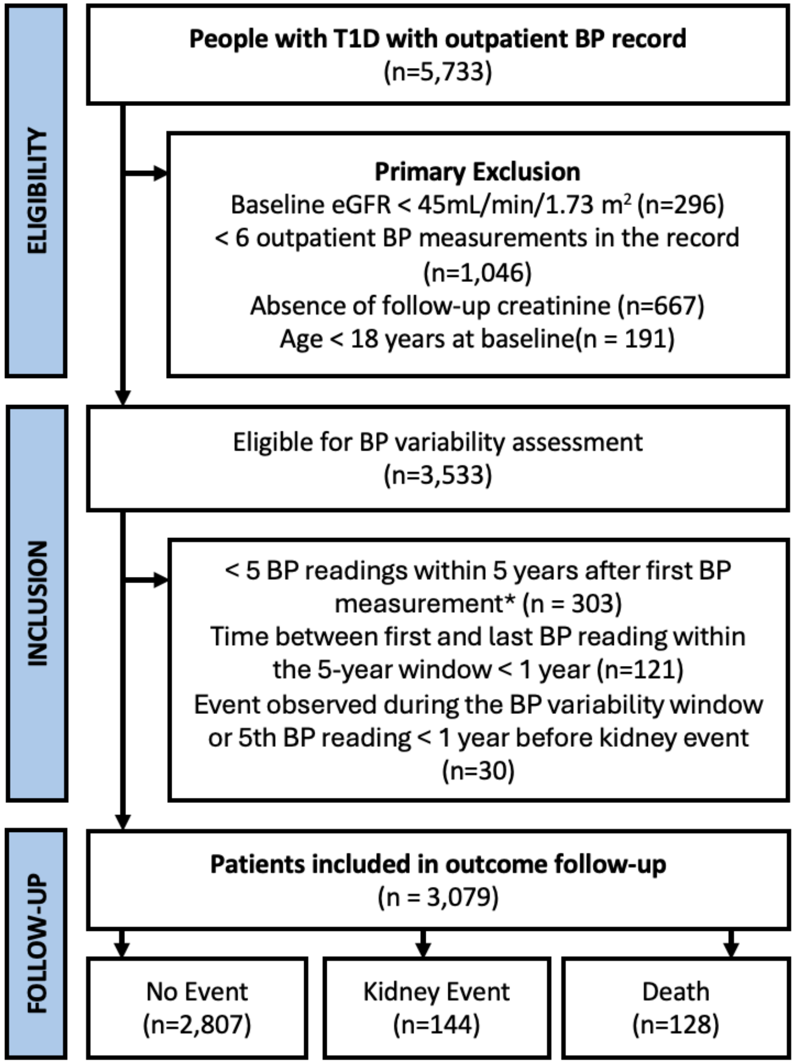

ESM Figure 2. Cumulative incidence plot of kidney endpoint and death as competing risk over 12 years

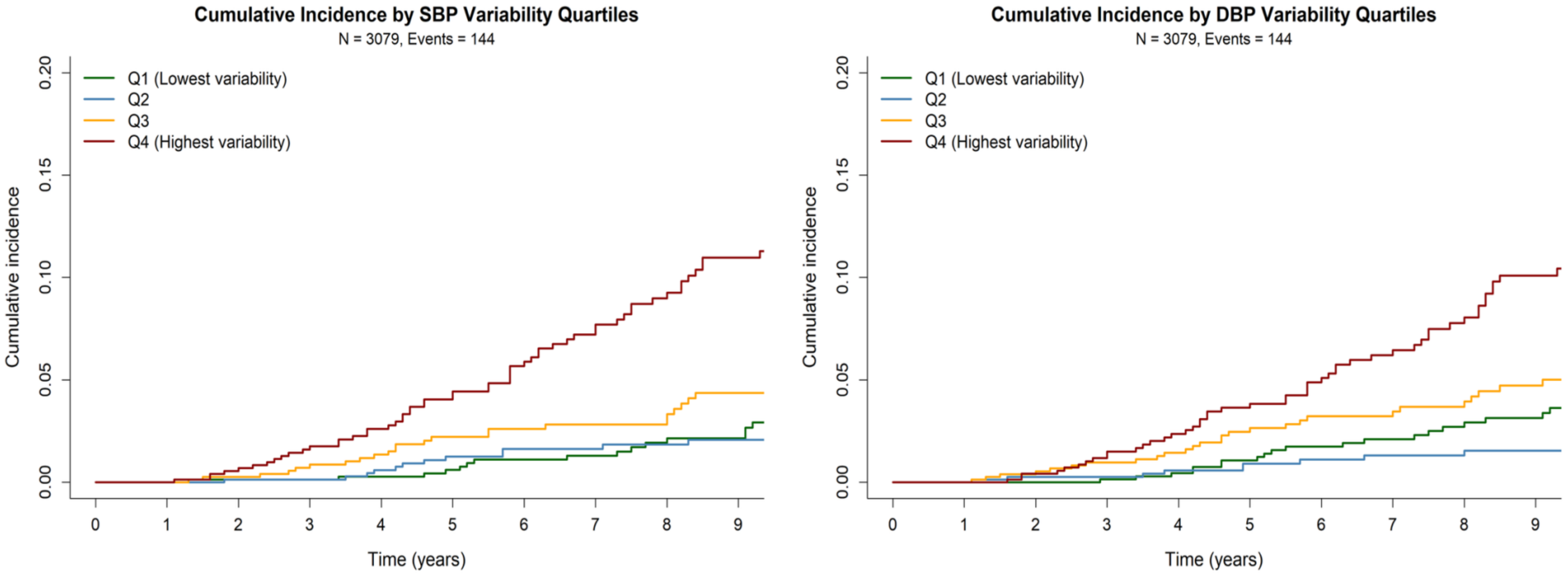

ESM Figure 3. Restricted cubic spline graphs

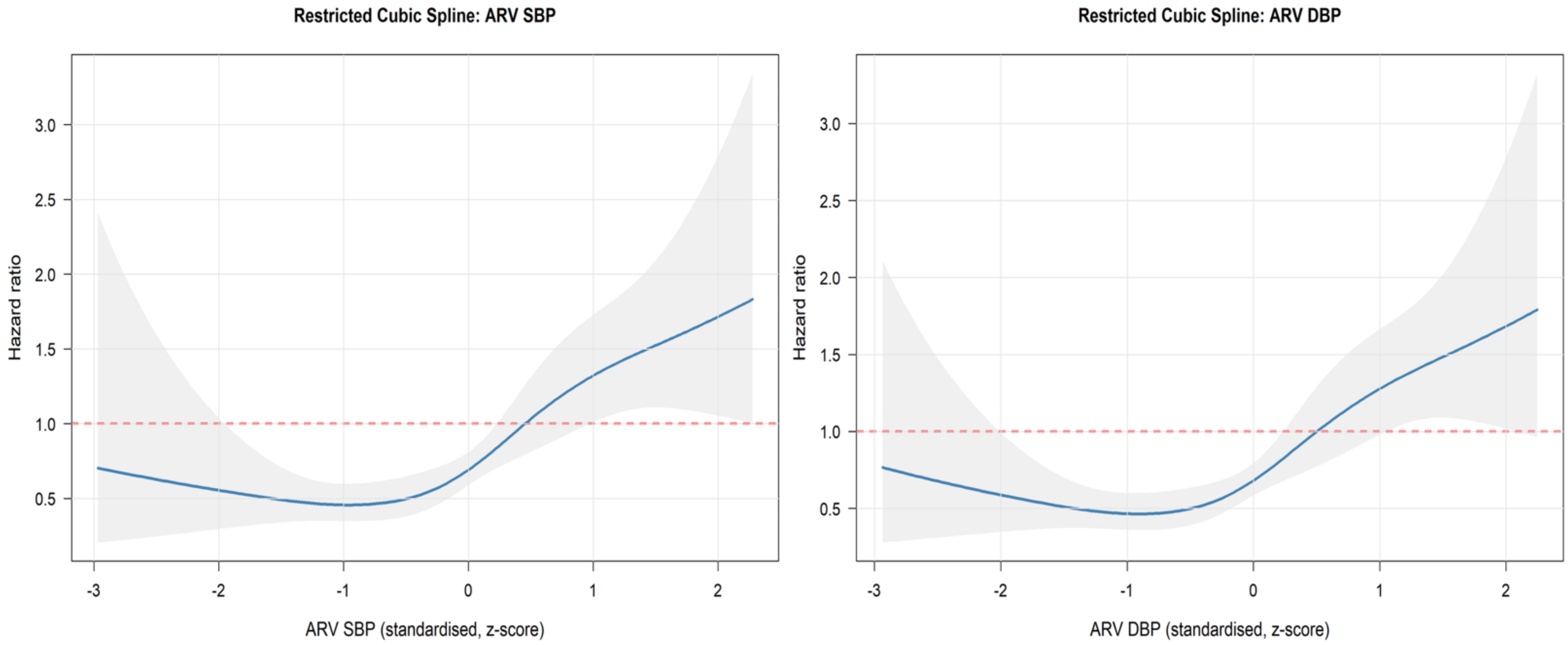

Supplement: Supplementary file 1 — ESM (PDF 644 KB) [file 125_2026_6715_MOESM1_ESM.pdf]
